# Upconversion Nanoparticle-Based Luminescent Aptasensor on a Porous Silicon Substrate for Isocarbophos Detection

**DOI:** 10.3390/s26165145

**Published:** 2026-08-14

**Authors:** Yangzhi Zhang, Xingyu Wang, Wu Le, Zhenhong Jia, Ziyi Yang, Xiaohui Huang, Jiajia Wang

**Affiliations:** 1 School of Physical Science and Technology, Xinjiang University, Urumqi 830046, China; 2 Xinjiang Space-Air-Ground Integrated Intelligent Computing Technology Laboratory, Changji 831100, China; 3 School of Information Science and Engineering, Xinjiang University, Urumqi 830046, China

**Keywords:** porous silicon, aptasensor, isocarbophos, upconversion fluorescence

## Abstract

Rare earth-doped upconversion nanoparticles (UCNPs) can emit upconversion luminescence under infrared light excitation and have been widely applied in the field of biosensing. In this study, upconversion nanoparticles were loaded onto porous silicon (PSi), and the detection of isocarbophos concentration was realized by virtue of nucleic acid aptamers. Firstly, the PSi was prepared by electrochemical etching and further functionalized. Nucleic acid aptamers were immobilized on the inner wall of PSi. Subsequently, the aptamer complementary strands labeled with UCNPs hybridized with the immobilized nucleic acid aptamers, enabling the indirect attachment of UCNPs to the inner wall of PSi. Then, isocarbophos solutions with different concentrations were added. The upconversion fluorescence images of samples before and after biological reaction were collected by an image acquisition device. The variation in average gray value of the images was obtained by image processing software, thereby achieving the quantitative detection of isocarbophos concentration. The limit of detection of this method for isocarbophos is 0.73 µg/L, which indicates that the proposed method can realize low-cost, high-sensitivity, and convenient biological detection of isocarbophos, with a fast and equipment-free signal readout.

## 1. Introduction

With the rapid development of modern agriculture, pesticides play an irreplaceable role in preventing and controlling diseases and pests, increasing crop yields and ensuring food security. However, the extensive application of pesticides has also caused serious pesticide residue problems [1]. Enriched through the food chain and agricultural water runoff, pesticide residues not only endanger the ecological balance, but also pose a direct threat to human life and health. In particular, the continuous contamination of environmental water bodies by pesticide runoff represents a critical primary stage of ecological exposure. Isocarbophos is a high-efficiency and broad-spectrum organophosphorus insecticide belonging to the thiophosphoramide class. It has been widely used in the pest and disease control of cotton, fruit trees, rice and other crops, and plays a vital role in agricultural production guarantee and crop yield improvement. Nevertheless, isocarbophos is a highly toxic pesticide that can induce neurological disorders. In severe cases, it may cause convulsions, respiratory failure and even death [2]. Most traditional pesticide residue detection methods rely on large analytical instruments with complicated operation and high cost, which are unsuitable for large-scale screening. To address these limitations, various novel detection strategies for isocarbophos have been developed in recent years. Among them, aptamer-based biosensors have garnered significant attention due to their high specificity. Recent studies have successfully integrated aptamers with multiple sensing modalities, including fluorescent [3,4] and colorimetric [5,6] assays, to achieve highly sensitive detection. The aptamer-recognition rationale is further supported by Tang et al.’s comprehensive review of nucleic acid aptamer-based sensors for bacteria detection, which highlights the versatility of aptamer design strategies across diverse target classes [7]. Moreover, Zhang et al. reported DNA-directed electrochemiluminescence nanospheres integrated with electrocatalysis-enhanced microfluidic arrays for rapid multibacterial detection, showcasing the potential of electrochemiluminescence as a sensitive, multiplexed sensing modality [8]. In addition, electrochemical sensors have proven effective for isocarbophos analysis by utilizing advanced nanomaterials or enzyme-inhibition mechanisms [9,10]. Beyond enzyme inhibition, nanostructured transducers have substantially broadened the electrochemical detection of pesticide and antibiotic residues; for example, Li et al. constructed an ultrasensitive fenitrothion sensor using MIL-125-derived Fe/Ti bimetallic oxide-doped porous carbon composites [11], and Xie et al. developed a nanoplate-like α-zirconium phosphate/carboxylated MWCNT voltammetric platform for trace norfloxacin determination [12], both demonstrating the potential of rationally designed nanocomposites for contaminant sensing. Notably, to further improve point-of-care and on-site testing capabilities, smartphone-based detection platforms have increasingly been incorporated into these sensing systems, allowing for rapid visual and quantitative readouts [4]. Despite these advancements, many of these methods still face challenges such as the need for multifaceted signal amplification, susceptibility to autofluorescence in complex matrices, or complicated modification processes. Therefore, it is highly relevant to develop an efficient, highly sensitive, background-free, and portable detection technology for monitoring pesticide levels in aqueous environments, which serves as a fundamental proof-of-concept step toward comprehensive agricultural and food safety.

UCNPs offer numerous advantages, including minimal damage to biological tissues, strong tissue penetration, absence of background fluorescence interference, and no photobleaching effects [13,14], and have been widely applied in fields such as biosensors [15,16,17] and bioimaging [18]. In the broader fluorescent-probe community, Men et al. reviewed activatable fluorescent probes for hepatocellular carcinoma imaging and diagnosis, demonstrating that stimuli-responsive signal activation can substantially improve sensing specificity and contrast [19]. To date, a large number of scholars have published articles on the application of UCNPs in biosensing technologies. For example, Ju et al. [20] used lanthanide-doped LiYF4 nanoparticles to propose a sandwich hybridization method based on a paper platform for the selective quantitative detection of DNA; Huang et al. [21] used NaYF4:Yb,Ho particles modified with anti-carcinoembryonic antigen (CEA) antibodies as probes and successfully detected CEA in human serum.

As a nanostructured material, PSi is widely applied in the field of biosensors due to its excellent properties, including a large specific surface area, good biocompatibility, controllable pore size, and easy functionalization [22,23,24,25]. Currently, PSi optical biosensors are mainly divided into two categories: those based on fluorescence variation [26,27,28] and those based on refractive index variation [29,30]. Among them, biosensors based on fluorescence variation achieve detection through fluorescence generated by the excitation of semiconductor or graphene quantum dots, with advantages of high sensitivity, good selectivity, low sample consumption, fast response, and excellent reproducibility. As a representative advance in ratiometric fluorescence sensing, Li et al. integrated a zirconium metal–organic cage with fluorescein sodium to construct a dual-emission platform for the ratiometric detection of sunset yellow, illustrating how dual-wavelength signal referencing can enhance quantitative reliability [31]. However, the excitation of conventional quantum dots requires short-wavelength light (typically less than 480 nm) or even ultraviolet light, which may exert certain adverse effects on the detected biomolecules. In addition, PSi strongly absorbs short-wavelength light, thereby reducing the excitation efficiency of short-wavelength light on the fluorescent particles inside PSi. In contrast, the use of near-infrared excitation light leads to minimal absorption in PSi, enabling more effective excitation of the fluorescent particles labeled with biological probe molecules in the PSi pores. Moreover, since the optical transmission window of biological tissues is located in the near-infrared region, the application of near-infrared excitation light causes less photodamage to biomolecules [32] and avoids the autofluorescence of many organisms and their tissues induced by visible light excitation—an interference factor for biodetection methods based on changes in the grayscale values of fluorescence images [33]. In summary, UCNPs excited by near-infrared light serve as an excellent fluorescent labeling material for PSi optical biosensors. Looking forward, the grayscale/image-based readout employed in this work could benefit from ongoing advances in intelligent image processing; Wang et al. proposed DABF-Net, a dual-branch attention-guided bidirectional feature enhancement network for infrared small-target detection [34], and Wei et al. developed FSINet, a robust feature separation and integration network for multiscale SAR object detection [35], both of which offer transferable methodologies for automating target recognition and quantitative analysis in future digital sensing platforms.

This work developed a novel PSi upconversion fluorescence biosensor, which ingeniously combines the high-specificity recognition capability of nucleic acid aptamers with the infrared excitation advantage of UCNPs, to achieve highly sensitive quantitative analysis of isocarbophos concentration. First, a PSi substrate with a pore size of 50 nm was prepared by electrochemical etching. The nucleic acid aptamer was immobilized on the pore surface via functionalization, and the UCNP-labeled complementary aptamer strand was then added to react with it, indirectly immobilizing the UCNPs on the pore surface. When isocarbophos solutions of different concentrations were introduced, the stronger specific affinity between isocarbophos and the aptamer triggered the detachment of the complementary strand, leading to a decrease in upconversion fluorescence intensity. The detection of isocarbophos concentration was realized by calculating the change in the average grayscale value of the upconversion fluorescence images before and after the reaction, achieving low-cost, high-sensitivity, and convenient biological detection with a fast signal acquisition process that requires no large-scale instruments.

## 2. Materials and Methods

### 2.1. Detection of Biological Reactions

**Figure 1:**
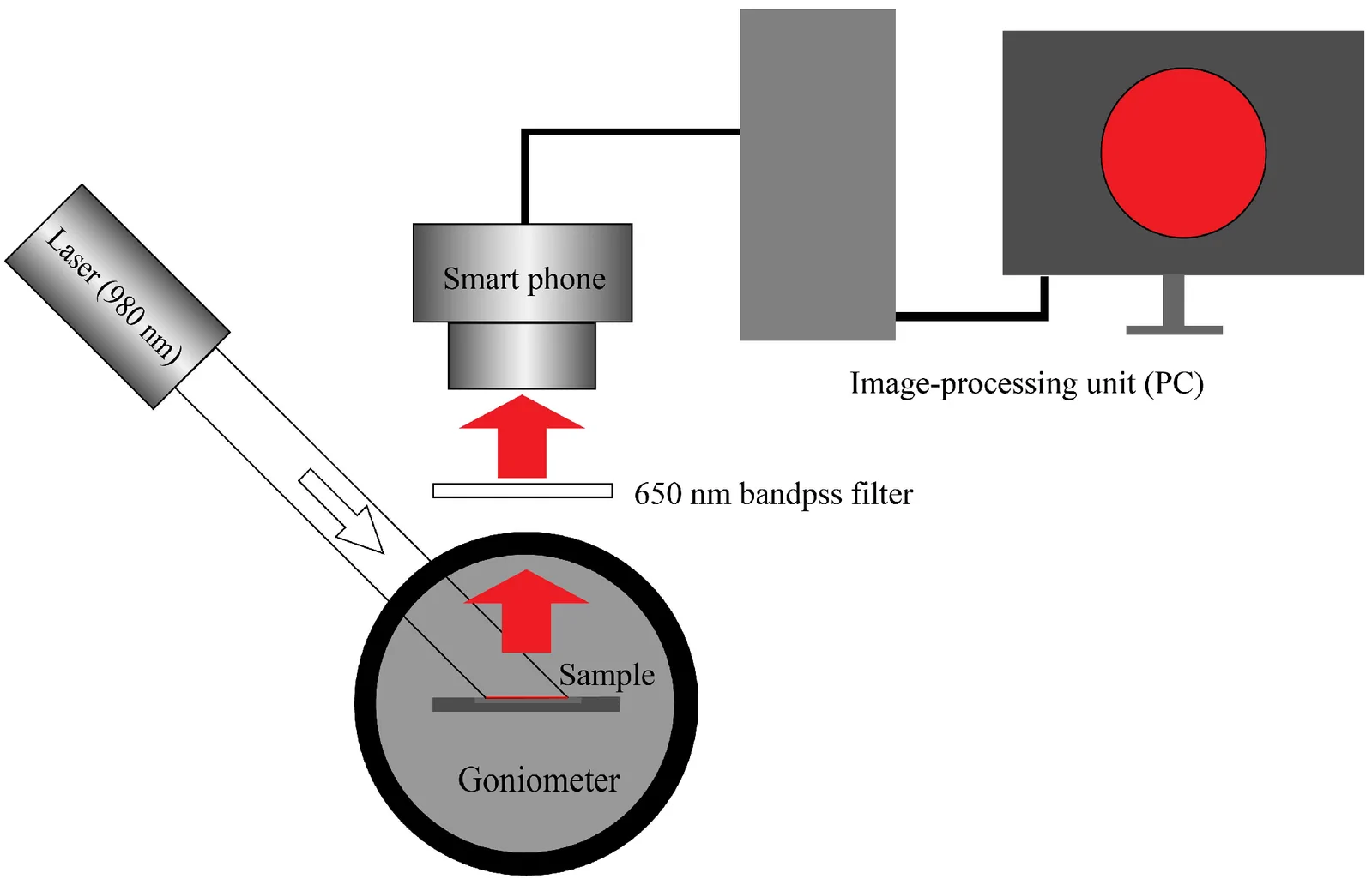
Biological reaction detection system.

This study achieves the detection of biological reactions by comparing the change in the average grayscale value of fluorescence images before and after the reaction. UCNPs are indirectly attached to the inner walls of the pores in PSi via the complementary strand of the aptamer. A 980 nm infrared laser irradiates the sample surface at a 45 angle, exciting the UCNPs within the pores to emit 650 nm upconversion fluorescence, which forms a red fluorescence image through the PSi surface. The imaging system captures these fluorescence images through a 650 nm bandpass filter. The change in the average grayscale value of the images before and after the biological reaction is then calculated using image processing software, enabling quantitative detection of the biological reaction. The detection system is illustrated in Figure 1. All imaging parameters were standardized throughout the study: 980 nm laser power (2 W), incident angle (45°), smartphone (iQOO 9 Pro) fixed at 10 cm from the sample, exposure time (0.1 s), ISO 800, and 650 nm bandpass filter (FWHM = 10 nm). A custom holder ensured consistent camera positioning.

### 2.2. Preparation of the PSi Substrate

**Figure 2:**
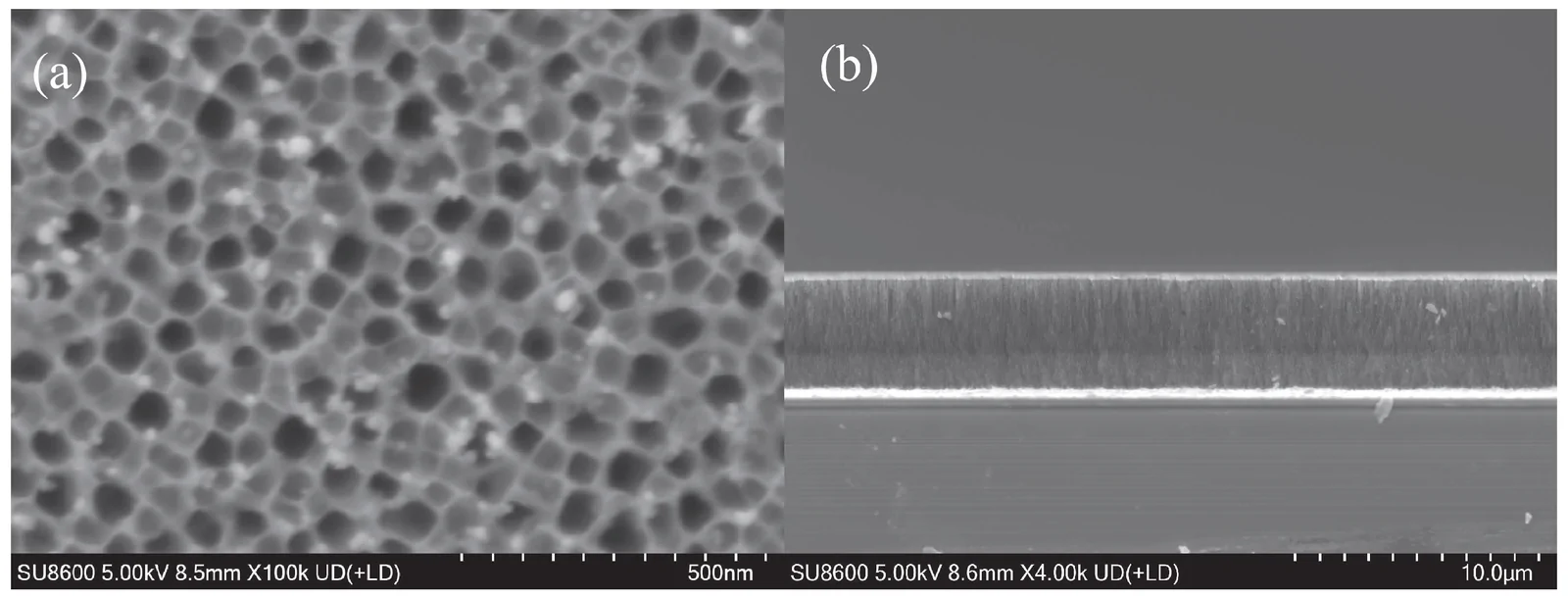
SEM images of the PSi substrate: (**a**) surface SEM image; (**b**) cross-sectional SEM image.

In this work, p-type monocrystalline silicon wafers (resistivity: 0.01–0.05 Ω·cm, crystal orientation: 〈100〉, thickness: 400 ± 25 µm) were used as the starting material. The wafers were first cut into 1.5 cm × 1.5 cm pieces, with a circular area of 0.8 cm in diameter defined as the region for PSi fabrication. The pieces were sequentially ultrasonically cleaned in acetone, ethanol, and deionized (DI) water for 10 min each to remove surface contaminants. The cleaned wafers were then placed in a custom-made polytetrafluoroethylene (PTFE) etching cell, into which a 1:1 (*v*/*v*) mixture of anhydrous ethanol and hydrofluoric acid (HF, 40%) was introduced. The etching parameters were controlled via LabVIEW software. The UCNPs used in this work were PAA-modified NaErF_4_:Yb/Tm@NaYF_4_, which were purchased from Hangzhou Xinqiao Biological Co., Ltd. (Hangzhou, China) In this core–shell structure, ${\mathrm{Yb}}^{3+}$ acts as the sensitizer to harvest 980 nm photons, while the heavily doped ${\mathrm{NaErF}}_{4}$ core induces cross-relaxation between ${\mathrm{Er}}^{3+}$ ions to predominantly yield 650 nm red emission. ${\mathrm{Tm}}^{3+}$ further modulates the energy transfer to maximize red fluorescence, and the inert ${\mathrm{NaYF}}_{4}$ shell minimizes surface non-radiative quenching. The nanoparticles have a diameter of approximately 12 nm. Under 980 nm excitation, their fluorescence emission peak is located at 650 nm. To ensure the successful infiltration of UCNPs into the PSi matrix, anodic etching was performed at a current density of 120 mA/cm^2^ for 30 s, yielding PSi substrates with a mean pore size of 50 nm. After etching, the samples were removed, rinsed thoroughly with anhydrous ethanol and DI water, and dried under a nitrogen flow. The surface and cross-sectional scanning electron microscopy (SEM) images of the as-prepared PSi substrate are presented in Figure 2.

### 2.3. Functionalization of PSi Substrates

To immobilize target DNA molecules onto the inner walls of PSi substrates, a series of surface modifications including oxidation, silanization, and glutaraldehyde treatment were conducted. As-prepared PSi substrates are chemically unstable and susceptible to spontaneous oxidation; thus, oxidation treatment was performed to enhance their stability. The PSi substrates were immersed in 30% (*v*/*v*) hydrogen peroxide solution and heated in a drying oven at 60 °C for 3 h. After oxidation, the substrates were rinsed with anhydrous ethanol and DI water, followed by drying under nitrogen flow. Subsequently, silanization was performed on the oxidized PSi substrates. The oxidized substrates were immersed in 4% (3-aminopropyl)triethoxysilane (APTES) solution (APTES:methanol:DI water = 1:10:10, *v*/*v*/*v*) for 1 h. Afterward, the substrates were rinsed and dried under nitrogen flow to graft APTES molecules onto the PSi surfaces. To form stable amino groups on the surfaces, the samples were heated in a vacuum drying oven at 100 °C for 10 min and then cooled naturally to room temperature. Finally, glutaraldehyde treatment was carried out. The silanized PSi substrates were immersed in 2.5% glutaraldehyde solution (50% glutaraldehyde stock:DI water = 1:19, *v*/*v*) for 1 h, rinsed with phosphate-buffered saline (PBS, pH 7.4) and DI water, and dried under nitrogen flow.

### 2.4. Immobilization of Aptamers on the Pore Walls of PSi

In this experiment, the isocarbophos aptamer and its complementary sequence were purchased from Sangon Biotech Co., Ltd. (Shanghai, China). The specific base sequences are as follows:

Isocarbophos aptamer: 5′-AGCTTGCTGCAGCGATTCTTGATCGCCACAGAGCT-3′-NH_2_ [36].

Complementary strand: 5′-GATCAAGAATCGCTGCAGCAAGCT-3′-NH_2_.

The aptamer was diluted to 10 μM using phosphate-buffered saline (PBS), and 50 μL of the diluted aptamer solution was dropped onto the surface of the functionalized PSi substrate via a pipette. The substrate was incubated in a thermostatic chamber at 37 °C for 2 h in the dark to allow for the nucleophilic addition reaction between the amino groups at the aptamer termini and the aldehyde groups on the pore walls of PSi, thereby achieving covalent immobilization through the formation of stable Schiff base bonds. After the reaction, the substrate was rinsed thoroughly with copious amounts of PBS to completely remove the unbound free aptamers, followed by drying under nitrogen flow.

Subsequently, the substrate was immersed in 3 M ethanolamine hydrochloride solution and incubated again at 37 °C for 2 h. This step aims to block the unreacted residual aldehyde groups on the pore walls via the amino groups in ethanolamine molecules, which can effectively seal these highly active sites and prevent non-specific adsorption of the UCNPs-labeled complementary strand in the subsequent step. After blocking, the substrate was removed, rinsed thoroughly with PBS, and dried under nitrogen flow for further use.

### 2.5. Determination of the Optimal Concentration of Aptamer Complementary Strand

To determine the optimal concentration of the aptamer complementary strand, we conducted a concentration optimization experiment. First, 50 µL of 4 mg/mL UCNPs were added to 90 µL of PBS and shaken for 10 min. Then, 30 µL of 0.01 M EDC solution and 30 µL of 0.01 M NHS solution were added, and the mixture was shaken for 10 min to activate the carboxyl groups. Subsequently, 200 µL of the aptamer complementary strand at different concentrations were added, resulting in final concentrations of 10 µM, 15 µM, 20 µM, 25 µM, and 30 µM, respectively. The mixture was then shaken in the dark for 10 h to allow for sufficient conjugation between the aptamer complementary strand and UCNPs. After the reaction, the solution was centrifuged at 10,000 rpm for 10 min. Although this centrifugation condition might not achieve 100% sedimentation of the 12 nm nanoparticles, it was sufficient to effectively separate the nanoconjugates from the unreacted free aptamer complementary strands, which would otherwise severely interfere with the sensing interface. The supernatant was discarded, and the purified precipitate was retained and collected, providing a sufficient amount of UCNPs-cDNA to fully saturate the subsequent biological reactions.

**Figure 3:**
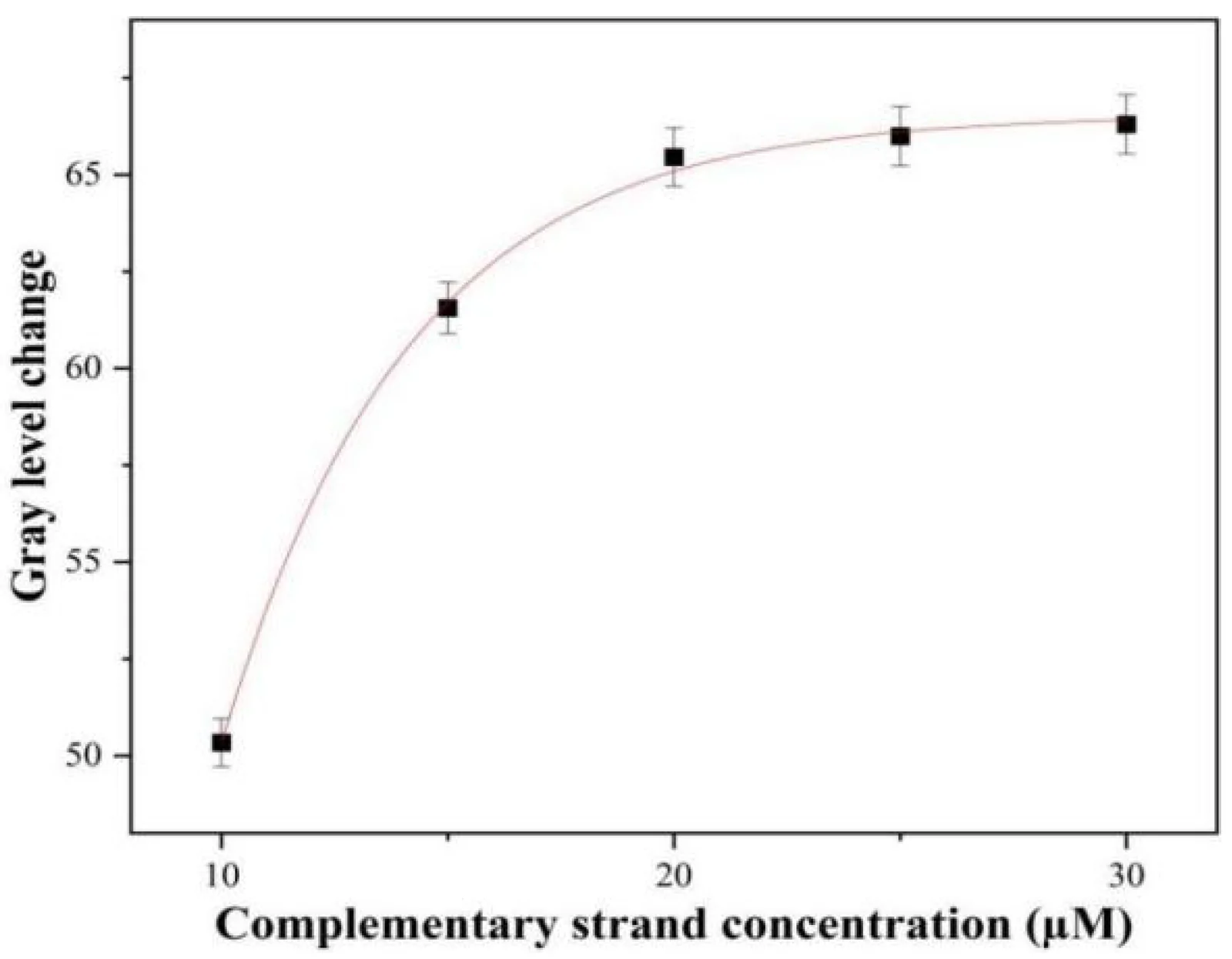
Aptamer Complementary Strand Concentration vs. Grayscale Value Change.

Using a pipette, 50 µL of each UCNPs-cDNA solution at different concentrations were added dropwise to the surface of the PSi substrate pre-functionalized with nucleic acid aptamers, and incubated at 37 °C for 2 h. After incubation, the substrate was rinsed with a large volume of PBS to remove UCNPs-cDNA that did not hybridize with the aptamers, and then dried under nitrogen. The aptamer complementary strand hybridizes with the nucleic acid aptamer via base pairing, thereby indirectly linking UCNPs to the pore walls of the PSi. The change in the average grayscale value of the fluorescence images of samples treated with different concentrations of UCNPs-cDNA, obtained using the biological reaction detection system, is shown in Figure 3.

The experimental results showed that when the concentration of the aptamer complementary strand ranged from 10 µM to 20 µM, the change in the average grayscale value of the images increased gradually with the increase in the concentration of the aptamer complementary strand. When the concentration of the aptamer complementary strand reached 20 µM, the change in the average grayscale value gradually became saturated and remained stable. Considering both the fluorescence signal intensity and reagent cost, the final concentration of the aptamer complementary strand used in subsequent experiments was set at 20 µM.

### 2.6. Quantitative Detection of Isocarbophos

The isocarbophos standard used in this experiment was purchased from Tanmo Quality Inspection Technology Co., Ltd. (Changzhou, China). To ensure the complete dissolution of isocarbophos and the preparation of an accurate standard stock solution, 0.005 g of isocarbophos powder was first weighed using an electronic balance. Then, 2 mL of acetone was added, and the mixture was ultrasonicated for 5 min to achieve complete dissolution. Subsequently, 48 mL of DI water was slowly added to bring the volume to the mark, resulting in a 100 mg/L isocarbophos standard stock solution.

**Figure 4:**
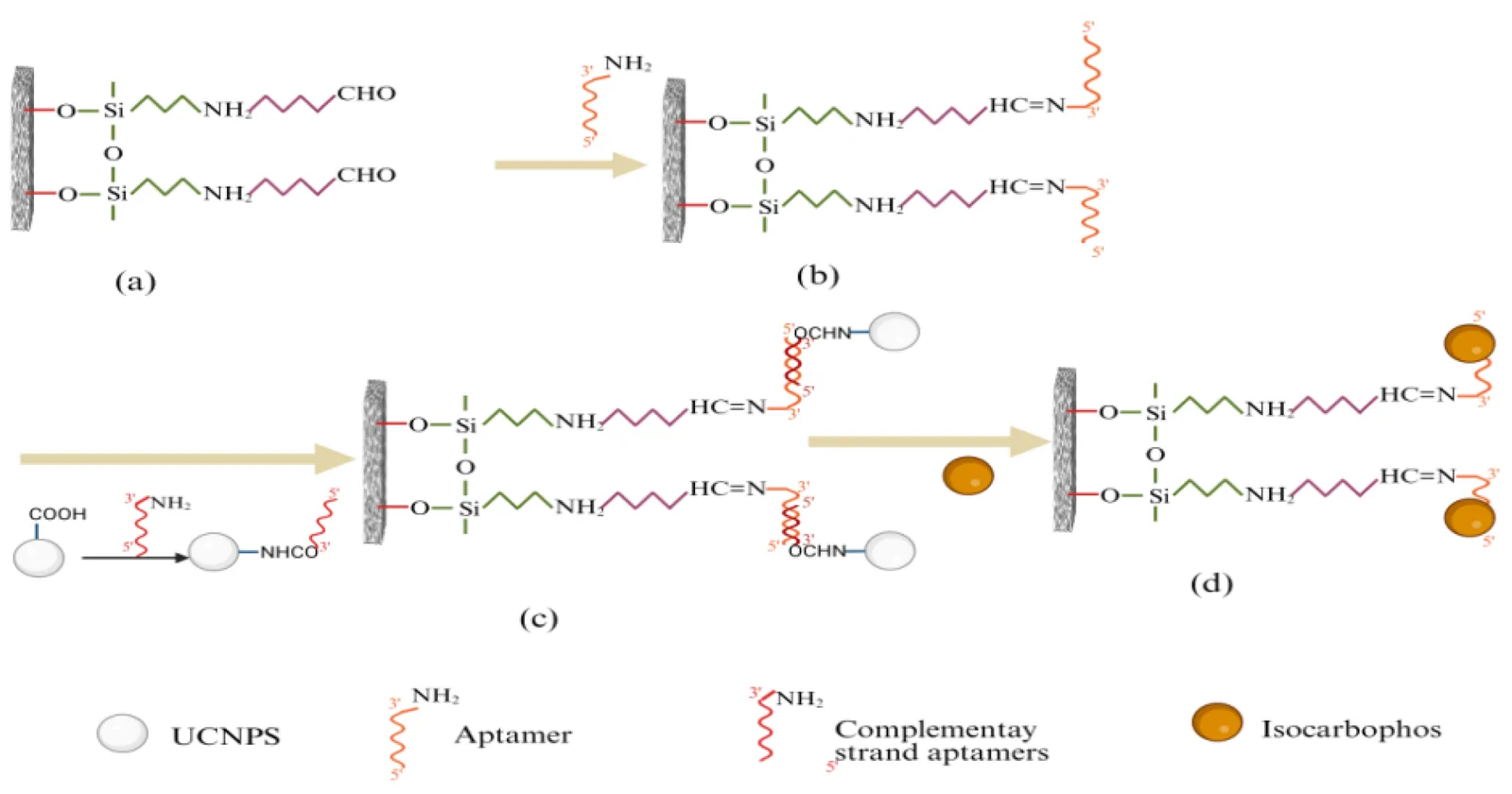
Schematic diagram of the biological reaction. (**a**) Glutaraldehyde-treated PSi; (**b**) Immobilization of nucleic acid aptamers on the pore walls of PSi; (**c**) Hybridization of nucleic acid aptamers with UCNPs-cDNA; (**d**) Coupling of isocarbophos with nucleic acid aptamers.

Prior to the actual detection, the above stock solution was serially diluted with DI water to obtain isocarbophos solutions with concentration gradients of 100 µg/L, 50 µg/L, 30 µg/L, 20 µg/L, 10 µg/L, and 5 µg/L. Using a pipette, 50 µL of isocarbophos solutions at different concentrations were added dropwise to the surface of the PSi substrate after reaction with UCNPs-cDNA, and the sample was incubated in a 37 °C incubator for 1 h [37]. Due to the stronger specific affinity of isocarbophos molecules for the nucleic acid aptamer, they preferentially bind to the aptamer on the pore walls, thereby forcing the UCNPs-cDNA molecules to dissociate from the double-stranded structure. After the reaction, the sample was rinsed with a large volume of PBS and dried under nitrogen. A schematic diagram of the biological reaction is shown in Figure 4. Subsequently, the upconversion fluorescence images before and after the reaction were acquired using the biological reaction detection system, and the change in the average grayscale value was calculated using image processing software.

## 3. Results and Discussion

### 3.1. Changes in Reflectance Spectra During the Functionalization Process

**Figure 5:**
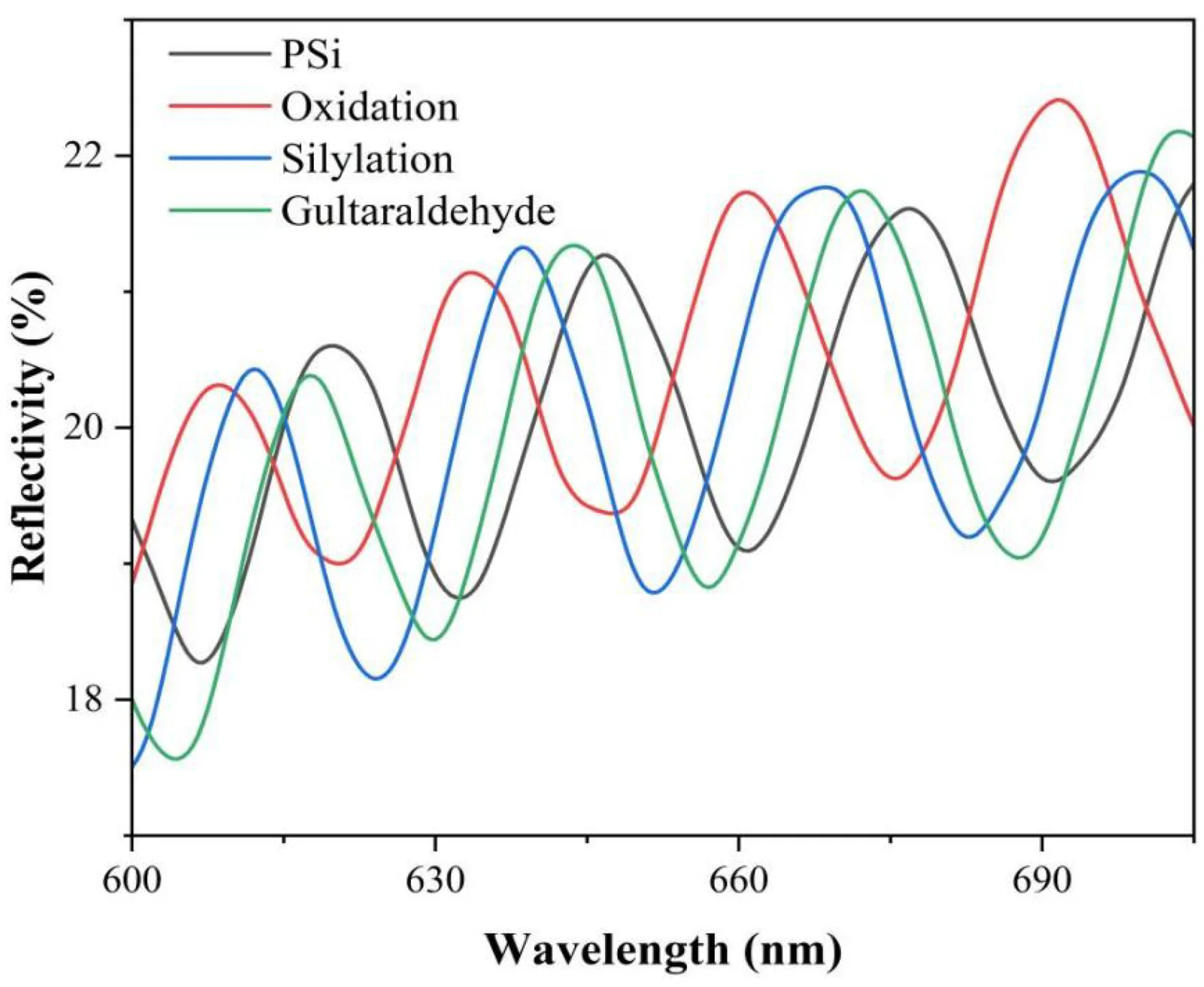
Changes in reflection spectra of PSi substrate during the functionalization process. Black line: freshly prepared PSi; red line: oxidized PSi; blue line: silanized PSi; green line: glutaraldehyde-treated PSi.

The functionalization steps, including oxidation, silanization, and glutaraldehyde treatment, all induce changes in the reflectance spectra of the substrate. Thus, the success of functionalization can be verified by analyzing these spectra. Figure 5 presents the reflectance spectra after each step: the black line corresponds to the freshly prepared PSi substrate, the red line to the oxidized substrate, the blue line to the silanized substrate, and the green line to the substrate after glutaraldehyde treatment. During oxidation, a SiO_2_ layer forms on the substrate surface. Since the refractive index of SiO_2_ (1.45) is significantly lower than that of monocrystalline silicon (3.4), a blue shift in the reflectance spectrum is observed. Subsequent silanization and glutaraldehyde treatment lead to the attachment of molecules to the pore walls, increasing the substrate’s effective refractive index and causing a red shift. The observed shifts in Figure 5 confirm that all functionalization steps were successfully completed.

### 3.2. Conjugation Characterization of UCNPs with Aptamer Complementary Strand

**Figure 6:**
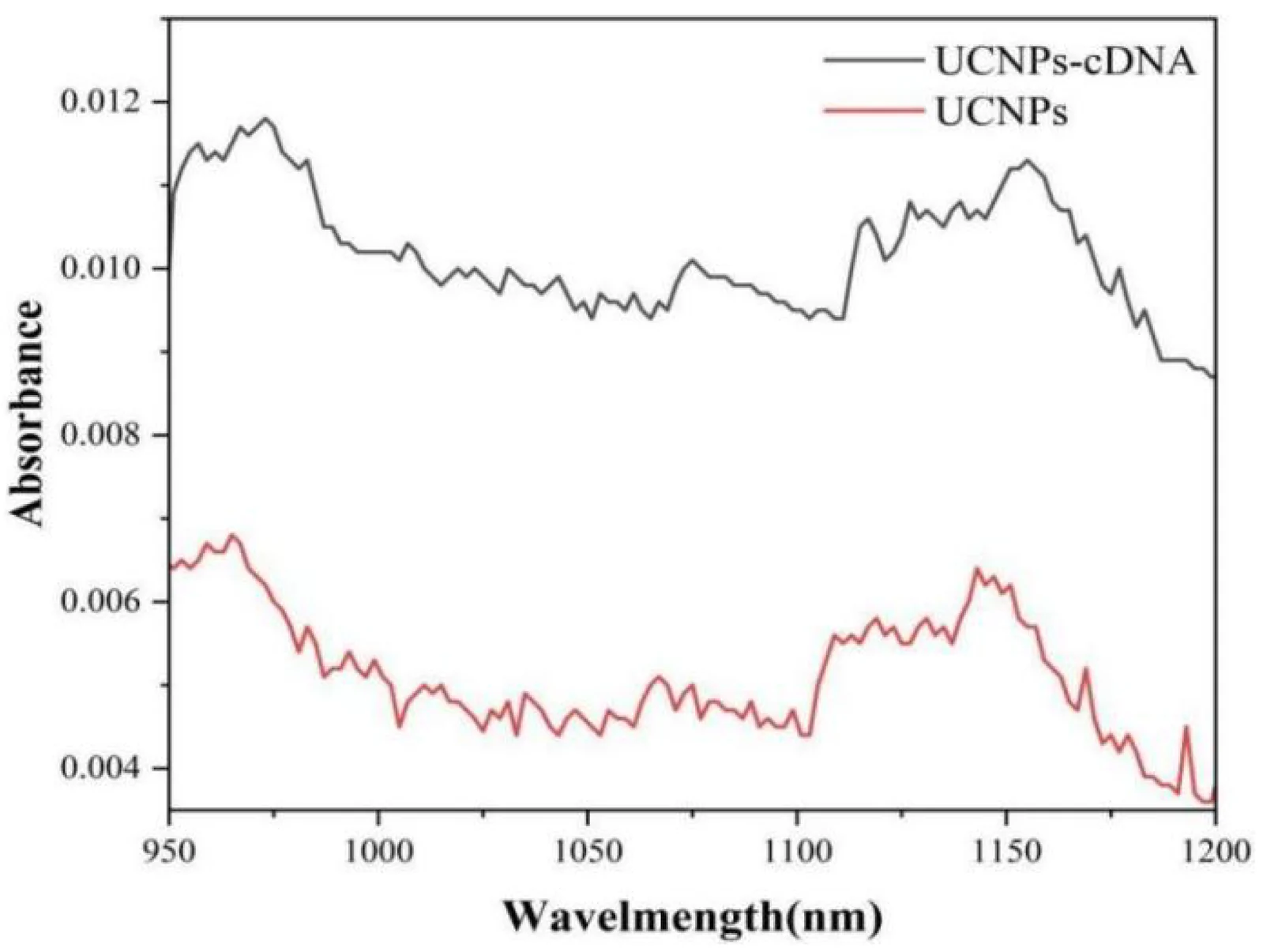
Absorption spectra of UCNPs and UCNPs-cDNA.

To verify whether UCNPs successfully formed covalent bonds with the aptamer complementary strand, this experiment characterized the nanoparticles before and after conjugation using UV-visible absorption spectroscopy. Figure 6 displays the absorption spectra of UCNPs and UCNPs-cDNA. The red line represents the absorption spectrum of UCNPs, while the black line corresponds to that of UCNPs-cDNA. The experimental data indicate that UCNPs exhibit distinct characteristic absorption peaks at 965 nm and 1143 nm in the near-infrared region. After conjugation with the aptamer complementary strand, the coating of DNA molecules significantly altered the local dielectric constant and the hydrophilic-hydrophobic microenvironment on the UCNP surface, causing the characteristic absorption peaks to shift to 973 nm and 1152 nm. This significant change in the absorption spectra provides supportive optical evidence indicating that the conjugation between UCNPs and the aptamer complementary strand has been successfully achieved.

### 3.3. Detection Results of Biological Reactions

**Figure 7:**
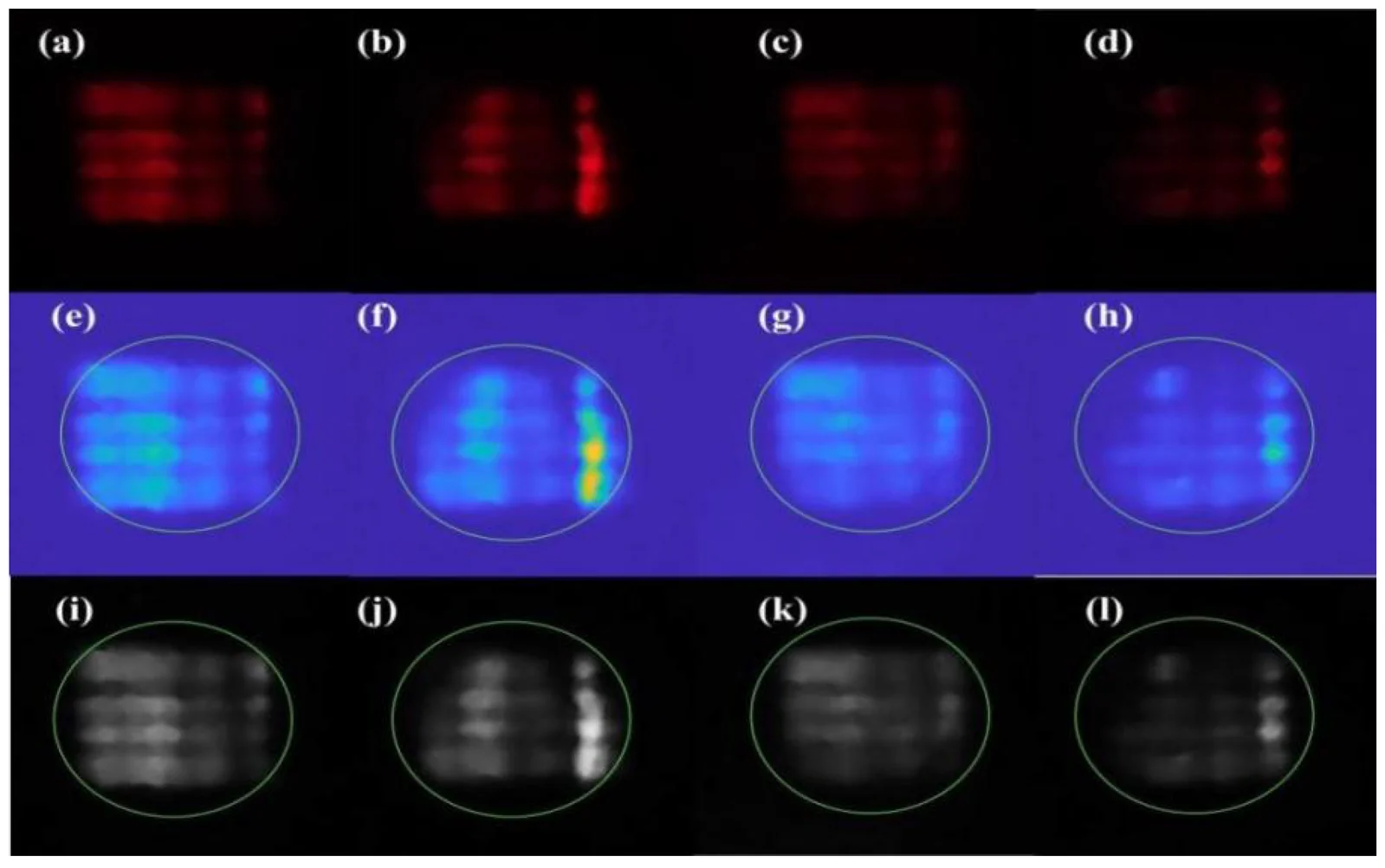
Upconversion fluorescence images of PSi substrate surface and their corresponding pseudo-color images and grayscale images. (**a**–**d**) Upconversion fluorescence images of the PSi substrate after reaction with DI water, 10 µg/L, 50 µg/L and 100 µg/L isocarbophos solution, respectively. (**e**–**h**) The corresponding pseudo-color images of (**a**–**d**). (**i**–**l**) The corresponding grayscale images of (**a**–**d**).

Figure 7a–d show the original upconversion fluorescence images acquired after adding DI water and isocarbophos solutions at concentrations of 10 µg/L, 50 µg/L, and 100 µg/L to a PSi substrate, followed by sufficient reaction. Figure 7e–h are the corresponding pseudo-color images, while Figure 7i–l are the corresponding grayscale images. The selected green circular region fully covers the entire laser spot irradiated on the PSi substrate surface, and the average gray value is calculated from the gray values of all pixels within this region. After conjugation of UCNPs-cDNA with the aptamer, in the absence of target isocarbophos solution, UCNPs-cDNA maintains a stable double-stranded hybridization state with the aptamer immobilized on the pore walls, resulting in the strongest upconversion fluorescence intensity. As the concentration of the added isocarbophos solution increases, the specific affinity between isocarbophos molecules and the aptamer is much higher than that between the aptamer and its complementary strand. Consequently, increasing amounts of UCNPs-cDNA are competitively displaced and fall off from the PSi pores, leading to a decrease in upconversion fluorescence intensity with increasing isocarbophos concentration.

**Figure 8:**
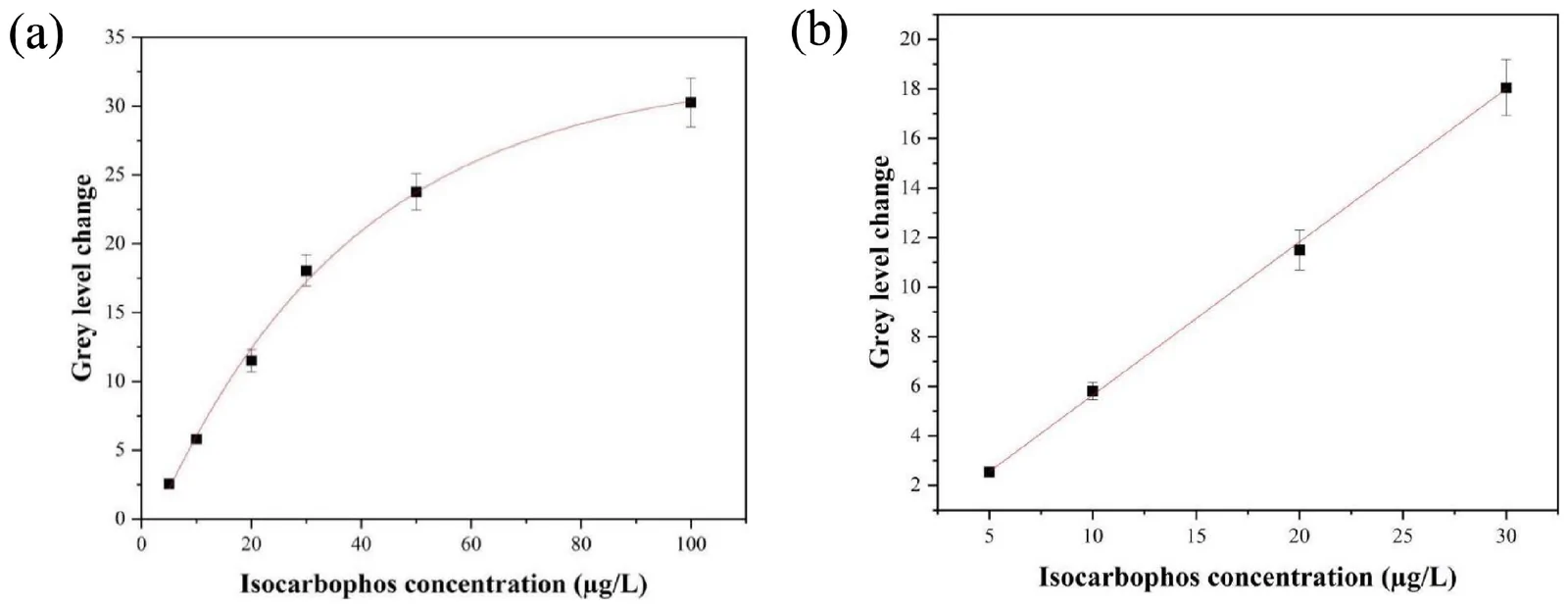
Relationship between isocarbophos concentration and the corresponding average grayscale value variation of fluorescence images before and after biological hybridization reaction, in which the curves represent the fitting curves. (**a**) 5 µg/L-100 µg/L; (**b**) 5 µg/L-30 µg/L.

Figure 8a illustrates the relationship between the addition of isocarbophos solutions with different concentrations ranging from 5 µg/L to 100 µg/L and the corresponding variation in the average grayscale value of upconversion fluorescence images. A good logarithmic relationship exists between the concentration of the added isocarbophos solution and the variation in average grayscale value within this range, and the logarithmic equation is

(1) $$Y=-34.95e^{-\frac{X}{3636}}+32.63$$

In Equation (1), *Y* represents the variation in the average grayscale value of the upconversion fluorescence image, and *X* denotes the concentration of the isocarbophos solution. The fitting correlation coefficient is 0.999.

In addition, within the concentration range of 5 µg/L to 30 µg/L, the concentration of isocarbophos solution exhibits a good linear relationship with the variation in the average grayscale value of its upconversion fluorescence images, as shown in Figure 8b. The linear equation is

(2) Y=0.62X−0.54

The fitting correlation coefficient of Equation (2) is 0.999. The limit of detection (LOD) was calculated to be 0.73 µg/L (2.52 nM) based on the 3σ/S criteria (where σ is the standard deviation of the blank samples (n=10) and *S* is the slope of the linear calibration curve).

### 3.4. Reproducibility and Stability

**Figure 9:**
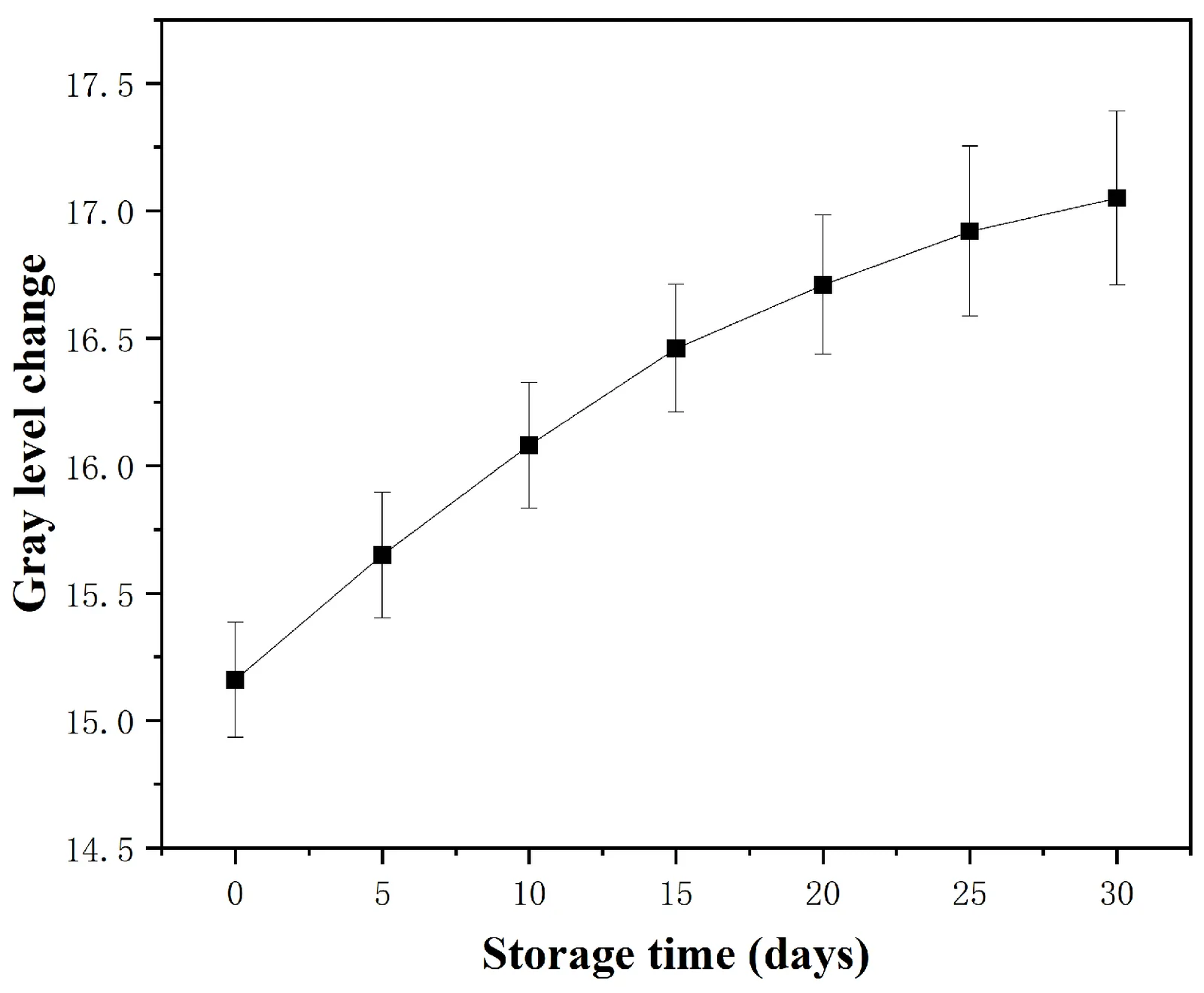
Changes in grayscale values of the sensor over 30 days of storage.

The stability and reproducibility of sensor detection are key factors for reliable target detection. In this experiment, 20 PSi substrates were prepared under identical conditions and tested with a 30 µg/L isocarbophos solution. The variations in the average grayscale values of these 20 samples were recorded, and the relative standard deviation was calculated to be 2.8%. Subsequently, the samples were stored at 4 °C in the dark under dry conditions, and their average grayscale values were measured every 5 days over a period of 30 days, as shown in Figure 9. The corresponding changes were recorded as 15.16, 15.65, 16.08, 16.46, 16.73, 16.92 and 17.05, respectively. The gradual decrease in grayscale values observed over 30 days may be attributed to minor decomposition of the upconversion nanoparticles or natural degradation of the aptamer and its complementary strand. Nevertheless, the sensor still retained 89% of its initial signal intensity after 30 days, demonstrating good long-term stability. These results confirm that the isocarbophos sensor prepared in this study exhibits excellent stability and reproducibility.

### 3.5. Advantages of Sensors Fabricated in This Study

To more intuitively evaluate the academic and applied value of this study in the field of isocarbophos detection, we performed a horizontal comparison of the performance of the proposed sensor with mainstream detection methods reported in recent years. The comparative data in Table 1 show that the detection limit of the UCNPs-based PSi biosensor constructed in this work is superior to that of label-free fluorescent sensors based on MWCNTs and G-quadruplexes [3], and is comparable to that of multicolor fluorescent aptasensors, C_60_OH trimodal sensors, and colorimetric–ratiometric dual-modal sensors [4,5,6]. However, these conventional high-sensitivity detection platforms often rely heavily on cumbersome and expensive large-scale optical analytical instruments.

In contrast, the most significant highlight and breakthrough of this study lies in eliminating the need for costly spectroscopic equipment; the image acquisition can be completed in just a few seconds using a smartphone-based portable imaging platform, which integrates a 980 nm laser for excitation, an optical filter, and a smartphone camera, followed by rapid signal readout via PC-based image processing software. Furthermore, the core innovation of this study does not rely solely on a single material or individual technology, but lies in the synergistic combination of the large specific surface area of the PSi substrate, the background-free fluorescence of UCNPs under near-infrared excitation, and portable digital image readout. This integrated platform successfully translates highly specific biomolecular recognition into quantifiable digital gray values, overcoming the reliance on traditional large-scale optical instruments and ultimately achieving a low-cost, rapid, and portable biological detection method for isocarbophos.

## 4. Conclusions

In this study, PSi substrates with a pore size of 50 nm were prepared via anodic electrochemical etching. The aptamer specific to isocarbophos was immobilized on the pore walls of the PSi, followed by hybridization with UCNPs-labeled complementary aptamer strands, which indirectly linked the UCNPs to the PSi surface. When isocarbophos solution was subsequently introduced, the target molecules, due to their higher specific affinity for the aptamer, competitively displaced the UCNPs-labeled complementary strands, resulting in a decrease in fluorescence intensity.

Upconversion fluorescence images were acquired before and after the addition of isocarbophos using an image capture device. The variation in the average grayscale value between these images was then calculated using image processing software, establishing a relationship between isocarbophos concentration and the change in average grayscale value. This enabled the quantitative detection of isocarbophos concentration. In summary, the key novelty of this work is the successful integration of the PSi substrate as a high-capacity carrier, UCNPs as background-free fluorescent labels, and portable digital image readout technology. The sensor developed in this study exhibits a low detection limit of 0.73 µg/L with high sensitivity, enabling low-cost and convenient biological detection with the advantage of a rapid signal readout. While the current study successfully validates the analytical performance and proof-of-concept of this integrated platform in standard aqueous solutions, future research will focus on optimizing sample pretreatment protocols to evaluate the sensor’s practical applicability in complex environmental water and real agricultural matrices.

## Figures and Tables

**Table 1:** Comparison of detection limits of isocarbophos sensors.

Sensor	Detection Limit	Reference
MWCNTs and G-quadruplex-based label-free fluorescent sensor	10 nM	[3]
Multicolor fluorescent aptasensor	3.01 nM	[4]
C_60_OH-based trimodal sensor	2 nM	[5]
Colorimetric–ratiometric dual-modal sensor	2.79 µg/L	[6]
UCNP-PSi aptasensor	0.73 µg/L	This work

## Data Availability

The data supporting the findings of this study are available from the corresponding author upon reasonable request.
